# Impact of type of vascular access on clinical outcomes in peritoneal dialysis patients transitioning to haemodialysis: an ANZDATA study

**DOI:** 10.1093/ckj/sfaf025

**Published:** 2025-01-25

**Authors:** Hicham I Cheikh Hassan, Karumathil Murali, Jenny H C Chen, Judy Mullan

**Affiliations:** School of Medicine, Lebanese American University, Beirut, Lebanon; Graduate School of Medicine, Faculty of Science, Medicine and Health, University of Wollongong, Wollongong, NSW, Australia; Graduate School of Medicine, Faculty of Science, Medicine and Health, University of Wollongong, Wollongong, NSW, Australia; Department of Renal Medicine, Wollongong Hospital, Wollongong, NSW, Australia; Graduate School of Medicine, Faculty of Science, Medicine and Health, University of Wollongong, Wollongong, NSW, Australia; Department of Renal Medicine, Wollongong Hospital, Wollongong, NSW, Australia; Graduate School of Medicine, Faculty of Science, Medicine and Health, University of Wollongong, Wollongong, NSW, Australia

**Keywords:** cause-specific mortality, haemodialysis, haemodialysis transfer, mortality, peritoneal dialysis

## Abstract

**Background:**

Type of vascular access used for haemodialysis is associated with long-term outcomes. However, the effect of access on haemodialysis transfer for peritoneal dialysis (PD) patients has not been fully explored.

**Methods:**

A retrospective cohort study was performed in incident adult PD patients from the Australian and New Zealand Dialysis and Transplant (ANZDATA) Registry who transferred to haemodialysis between 2004 and 2022. Associations between vascular access on transfer [central venous catheter (CVC) or arterio-venous access (AVA)] and clinical outcomes (all-cause mortality, cause-specific mortality, kidney transplantation and return to PD) were compared using Cox proportional hazards analysis and competing risk models.

**Results:**

Of 6824 patients, 65% used a CVC on transfer and 35% an AVA. Variability of access type at transfer between centres was high (range 13%–98% for CVC). AVA transfer was associated with a longer PD vintage (1.6 versus 1.2 years, *P *< .001) and inadequate PD as a cause of transfer (29% versus 15%, *P *< .001). All-cause mortality was lower for AVA transfer compared with a CVC [hazard ratio (HR) 0.71, 95% confidence interval (CI) 0.66–0.77]. The risk was lowest for infection-related mortality (HR 0.59, 95% CI 0.45–0.77) Kidney transplantation was more likely in AVA transfer compared with a CVC (HR 1.18, 95% CI 1.05–1.33), but return to PD was less likely (HR 0.67, 95% CI 0.59–0.71). Results remained consistent in the competing risk analysis.

**Conclusions:**

Patients who transferred with an AVA, compared with a CVC, showed better survival and kidney transplantation rates, but were less likely to return to PD.

KEY LEARNING POINTS **What was known:**

- The use of an arterio-venous fistula (AVF) as a vascular access is associated with better outcomes compared with a central venous catheter (CVC) for patients who start haemodialysis. However, these studies have only examined this association in patients who started haemodialysis as the first dialysis modality. Whether these results can be generalized to other patient populations, such as peritoneal dialysis (PD) patients who transfer to haemodialysis, has not been shown before.

**This study adds:**

- We examined long-term outcomes for patients who transfer to haemodialysis from PD with an AVF compared with a CVC, examining all-cause mortality and cause-specific mortality. There was a large variability of vascular access for haemodialysis transfer, ranging from CVC use in 13% of patients to 98% of patients according to centre. All-cause mortality was lower in patients who transferred with an AVF compared with a CVC, with the lowest risk for infection-related mortality.

**Potential impact:**

- Similar to patients who commenced haemodialysis as the first dialysis modality, PD patients who transfer with an AVF to HD have better overall survival rates compared with a CVC. The findings can be used to aid the decision-making process on the most suitable vascular access for haemodialysis transfer.

## INTRODUCTION

Peritoneal dialysis (PD) is one of the preferred treatment options for patients with kidney failure. PD offers residual kidney function preservation [1, 2], treatment schedule flexibility, quality of life maintenance, and improved mortality outcomes compared with facility haemodialysis (HD) [3, 4]. Despite these advantages PD still has limitations, including HD transfer. HD transfer can occur in almost 20% of PD patients in the first year alone [5, 6], and can affect 40%–50% of patients who start PD [7–9]. The most common cause of HD transfer is infection, with peritonitis the leading site of infection [6–8].

For HD patients the ideal vascular access is an arterio-venous access (AVA), as highlighted by initiatives such as the ‘fistula first approach’ [10]. HD patients who commence with an AVA have a lower risk of mortality and bloodstream infections and achieve better blood flow rates and solute clearance than HD patients starting with a central venous catheter (CVC) [10]. However, an AVA requires planning and preparation to allow time for maturation prior to use. This limits its practicality as the initial access during acute transfer from PD to HD. Maturation failure rates can also be high and range from 30% to 60% in the HD cohort [11, 12].

The optimal access for PD patients who transfer to HD has not been determined. The current evidence of AVA benefits over CVC is derived almost exclusively from incident HD patients, with the assumption that this evidence can be generalized and applied to the PD patient population. While an AVA may be the preferred vascular access, there are challenges specific to the PD population. For instance, the abrupt nature of some HD transfers for PD patients, especially during acute infections such as a peritonitis, does not allow sufficient time for AVA creation and maturation, while creating a pre-emptive AVA, often termed a backup arterio-venous fistula, risks subjecting patients to interventions that may not ultimately be needed.

To address this gap in the literature, a comprehensive study using a bi-national dialysis registry was conducted. We aimed to compare outcomes between PD patients who transfer to HD with an AVA versus CVC in terms of their (i) annual proportion and trends over time, (ii) all-cause mortality, (iii) cause-specific mortality, (iv) kidney transplantation, and (v) return to PD.

## MATERIALS AND METHODS

### Study design and participants

This retrospective cohort study analysed de-identified data from the Australia and New Zealand Dialysis and Transplant Registry (ANZDATA). It encompassed all adult patients on PD (≥18 years of age) in Australia and New Zealand, who commenced PD between 1 January 2004 and 31 December 2022, and who subsequently transferred to HD. We excluded patients who recovered kidney function or lacked data on vascular access used at the time of HD transfer. The study was approved by the research ethics committee (ISLHD/LNR/2023–204) at Illawarra and Shoalhaven Local Health District, Australia, and permission to use the data was granted by the ANZDATA executive. The study was conducted in accordance with the Strengthening the Reporting of Observational Studies in Epidemiology (STROBE) guidelines [13].

### Data collection

Baseline sociodemographic and clinical characteristics were collected at the time of HD transfer. These included gender, age, primary kidney disease (diabetic kidney disease, reno-vascular disease, glomerulonephritis, cystic disease, and other), body mass index (underweight, normal, overweight, and obese), smoking status (non-smoker, ex-smoker, and current smoker), comorbid conditions (chronic lung disease, coronary artery disease, cerebrovascular disease, peripheral vascular disease, diabetes mellitus, and cancer) and peritonitis episodes. Late referral to nephrologist was defined as referral to a nephrologist <3 months prior to PD initiation. PD vintage was categorized on time periods: ≤6 months, >6 to 24 months, and >24 months. Reasons for transitioning to HD transfer were classified as: infection-related causes (e.g. peritonitis, PD catheter-related infection, or any other infection); inadequate PD (i.e. inadequate ultrafiltration, solute clearance, or multiple adhesions); patient-related factors (such as inability to cope or self-care, patient preference, or poor nutrition); abdominal wall complication defects (e.g. hernia, scrotal oedema, or pleural leak); issues relating to abdominal surgery (due to elective or emergency abdominal surgical procedures); catheter-related problems (e.g. malpositioning, non-functioning, dialysate leak due to catheter, catheter blockage, or dislodgement of the catheter or cuff); and other/unspecified reasons.

### Exposure

The exposure variable of interest was the type of vascular access used during the first HD session, categorized into CVC (tunnelled and non-tunnelled) or AVA (natural or graft) access. We did not further subcategorize access due to small numbers of patients with an AVA graft (*n* = 103, 1.5%) or a non-tunnelled CVC (*n* = 625, 9%). We only considered HD access at first use.

### Outcome

The study start date was defined as the date of the initial transfer from PD to HD. The primary outcome was all-cause mortality. Secondary outcomes included cause-specific mortality, categorized as cardiovascular-related mortality, infection-related mortality, mortality due to withdrawal from treatment, and other causes of mortality. Cardiovascular-related mortality was defined as death assumed, suspected or attributable to a cardiac cause, such as ischaemic heart disease, heart failure or arrhythmia, based on predefined definitions according to ANZDATA (available at: https://www.anzdata.org.au/anzdata/services/data-management/data-forms/). Infection-related mortality was defined as death resulting from bacterial, viral, or fungal infection. In addition, we assessed kidney transplantation and return to PD as an outcome after HD transfer, based on the type of vascular access used at the initial HD session. Patients were followed up from the start date of HD transfer until the occurrence of death, transplant, or return to PD (as per outcome examined), loss of follow-up, or the end of the study period (31 December 2022). A sensitivity analysis was performed examining patients who remained on HD after transfer for >30 days and >90 days after their transfer. This analysis was undertaken because evidence from Australia suggests that up to 25% of patients ultimately resumed PD [14] within the first 30 days of transfer, and a further 18% within 60 days [15]. It was anticipated that including such patients would account for a bias, since these patients were more likely to use a CVC for temporary HD due to acute causes before returning to chronic PD.

### Statistical analysis

Categorical data were expressed as numbers (percentages) and continuous data as means with standard deviations (SD) or medians with interquartile range as per distribution. Baseline characteristics between groups was compared using the χ^2^ test, *t* test or Mann–Whitney *U* test as appropriate. The annual proportion of access type at first HD use was calculated according to CVC or AVA use. Annual trends were examined using a Jointpoint regression model [16] to estimate the annual percentage change. The association between the type of vascular access used during the first HD on HD transfer and patient outcomes was analysed by Cox proportional hazards regressions, with the model including a shared frailty term to account for vascular access variation between dialysis centres, to determine hazard risk (HR) and 95% confidence interval (95% CI). The following four models were constructed: Model 1, unadjusted; Model 2, Model 1 + baseline sociodemographic, demographic, and clinical characteristic variables (age, gender, BMI category, PD vintage, history of peritonitis, late referral, smoking status, and cause of kidney failure); Model 3, Model 2 + comorbid conditions (chronic lung disease, coronary artery disease, peripheral vascular disease, diabetes mellitus, or cancer); and Model 4, Model 3 + cause of HD transfer.

Fine–Gray extensions of the Cox proportional hazards model were used to fit competing risk regression models and featured a robust variance estimator to account for variations across treatment centres. For all-cause mortality, kidney transplantation was considered as a competing risk. For cause-specific mortality, both kidney transplantation and other causes of mortality were considered as competing events. For the outcome of return to PD, both kidney transplantation and mortality were used as competing events, while for kidney transplantation outcomes mortality was considered as a competing event. Results were expressed as sub-hazard ratios (SHRs) with 95% CI. No imputation of missing variables was attempted since the overall number of missing variables was low (<1% for late referral, BMI, smoking status, and cancer, and <0.5% for kidney failure cause, chronic lung disease, peripheral vascular disease, cerebrovascular disease, and diabetes). Sensitivity analyses were performed excluding patients who remained on HD <30 days and <90 days. Finally, potential effect modification of the relationship between vascular access and gender, PD vintage, previous peritonitis, cause of kidney failure, and cause of haemodialysis transfer was assessed by adding vascular access X tested variable interaction to models for all-cause mortality, transplantation, and return to PD.

Statistical analysis was performed using STATA 15.1 (StataCorp, College Station, TX) and Jointpoint (version 4.9.0.0). *P* values of <.05 were considered statistically significant.

## RESULTS

### Study population

**Figure 1:**
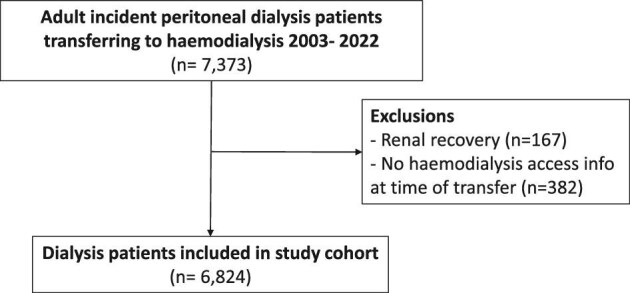
Patient flowchart for study cohort.

A total of 6824 incident patients on PD who transferred to HD were included in the study, after excluding patients with recovery of kidney function (*n* = 167) or unknown vascular access at the time of haemodialysis transfer (*n* = 382) (Fig. 1). Our cohort was majority male (62%) with a mean age of 61 (SD 14) years. The most common cause of kidney failure was diabetic kidney disease (39%), followed by glomerulonephritis (23%) (Table 1). Patients were on PD for a median of 1.3 (0.5, 2.6) years before transferring to HD. The main reasons for transferring to HD were infection-related causes (38%) and inadequate PD (20%).

**Table 1:** Baseline characteristics of all study group and by first vascular access at haemodialysis transfer.

		Vascular access	
	All study group *n* = 6824	AVA *n* = 2424 (35%)	CVC *n* = 4400 (65%)
Gender (male)	4199 (62)	1627 (67)	2572 (59)
Age (years)	61 (15)	61 (15)	61 (15)
Cause of kidney failure			
Diabetic kidney disease	2658 (39)	859 (36)	1799 (41)
Reno-vascular	941 (14)	330 (14)	611 (14)
Glomerulonephritis	1583 (23)	641 (27)	942 (22)
Cystic	550 (8)	211 (9)	339 (8)
Other	1076 (16)	376 (16)	700 (16)
Late referral to nephrologist	736 (11)	223 (9)	513 (12)
Smoking status			
Non-smoker	3160 (47)	1127 (47)	2033 (47)
Ex-smoker	2742 (41)	969 (40)	1773 (41)
Current smoker	871 (13)	304 (13)	567 (13)
BMI category			
Underweight	140 (2)	40 (2)	100 (2)
Normal	1 917 (28)	659 (27)	1 258 (29)
Overweight	2 354 (35)	846 (35)	1 508 (35)
Obese	2 361 (35)	863 (36)	1 498 (34)
Comorbidity			
Chronic lung disease	1085 (16)	357 (15)	728 (17)
Coronary artery disease	2140 (31)	756 (31)	1384 (32)
Peripheral vascular disease	1601 (24)	482 (20)	1119 (26)
Cerebrovascular disease	975 (14)	310 (13)	655 (15)
Diabetes mellitus	2656 (39)	875 (36)	1781 (41)
Cancer	1008 (15)	414 (17)	594 (14)
PD vintage (years)	1.3 (0.5, 2.6)	1.6 (0.7, 2.8)	1.2 (0.5, 2.4)
PD vintage			
≤6 months	1872 (27)	523 (22)	1349 (31)
>6–24 months	2596 (38)	922 (38)	1674 (38)
>24 months	2356 (35)	979 (40)	1377 (31)
Prior PD peritonitis	3961 (58)	1259 (52)	2702 (61)
Cause of HD transfer			
Infection-related	2563 (38%)	690 (29%)	1873 (43%)
Inadequate PD	1363 (20%)	704 (29%)	659 (15%)
Patient factors	823 (12%)	361 (15%)	462 (11%)
Abdominal wall defects	480 (7%)	131 (5%)	349 (8%)
Abdominal surgery	397 (6%)	122 (5%)	275 (6%)
Cather-related problems	624 (9%)	174 (7%)	450 (10%)
Unknown/other	574 (8%)	242 (10%)	332 (8%)

Data are expressed as number (percentage), mean (standard deviation) or median (intraquartile range).

### Access at haemodialysis transfer

**Figure 2:**
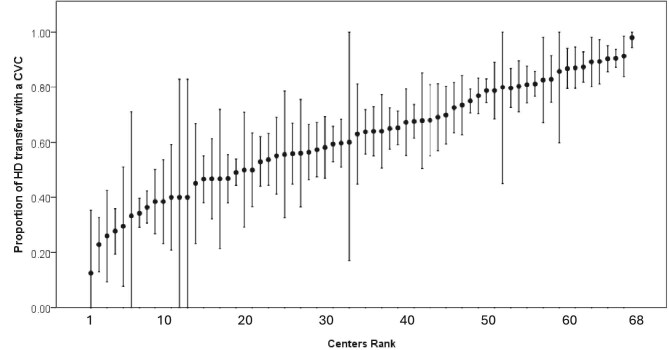
Variation of proportion of PD patients who transferred to HD with a CVC across 68 Australian and New Zealand dialysis centres during the period 2004–22. Circles represent the proportion while bars represent the 95% confidence interval. Centres with <10 patients were excluded.

**Figure 3:**
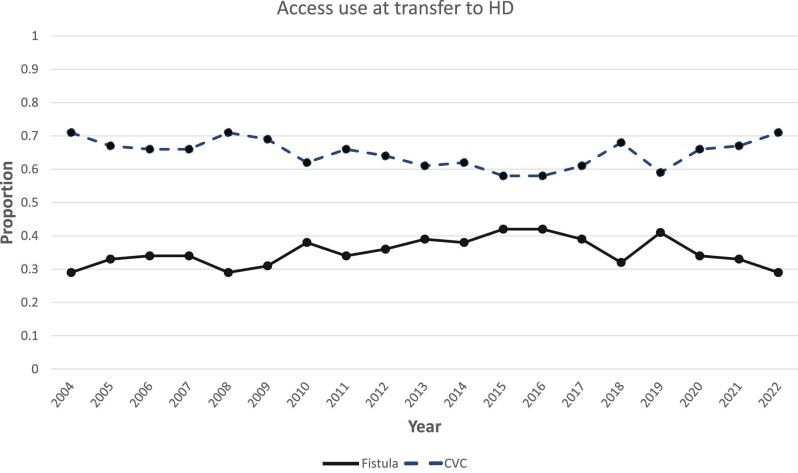
Annual proportion of access use at transfer to HD (fistula or CVC) from 2004 to 2022.

At HD transfer, the most common vascular access used was CVC compared with an AVA (65% versus 35%). A tunnelled CVC was used in 3775 (55%) patients, a non-tunnelled CVC in 625 (9%) patients, native AVA in 2321 (34%) patients, and a graft in 103 (1.5%) patients. Access type at HD transfer variability was high between centres, with CVC use ranging from 13% to 98% (Fig. 2). Patients transferring to HD with an AVA compared with a CVC were more likely to have a longer PD dialysis vintage (1.6 versus 1.2 years, *P *< .001) or inadequate PD as the cause of HD transfer (29% versus 15%, *P *< .001), and were less likely to have infection as a cause of HD transfer (29% versus 43%, *P *< .001) (Table 1). Over the study period, there was no change found in the proportions of vascular access type used at HD transfer [AVA annual percentage change (APC) 0.3, 95% CI −1.2 to 2.2, CVC APC −0.1, 95% CI −0.7 to 0.7] (Fig. 3).

### Association of access at haemodialysis transfer with all-cause mortality and cause-specific mortality

**Figure 4:**
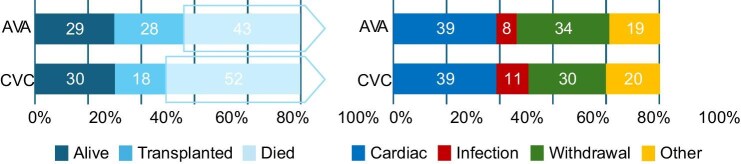
Bar graphs showing outcomes after transfer to HD stratified by vascular access type. For the outcome of death, the proportion of deaths from cardiovascular-related, infection-related, withdrawal, and other causes are shown.

Patients were followed up for a total of 21 096 patient-years after transfer to HD, with a median follow-up time of 2.3 years (IQR 0.9, 4.4). During follow-up, death occurred in 3342 (49%) patients, kidney transplant in 1481 (22%), and loss of follow-up in 14 (0.2%) patients. Causes of death were cardiovascular-related in 1308 (39%) patients, withdrawal from treatment-related in 1032 (30%) patients, infection-related in 333 (10%) patients, and other causes in 669 (20%) patients. The leading causes of death were similar in the two groups, with cardiovascular-related (39% for CVC and AVA) being the most common followed by withdrawal from treatment-related (30% for CVC, 34% for AVA), other causes (20% for CVC, 19% for AVA), and infection-related (11% for CVC, 8% for AVA) (Fig. 4).

**Figure 5:**
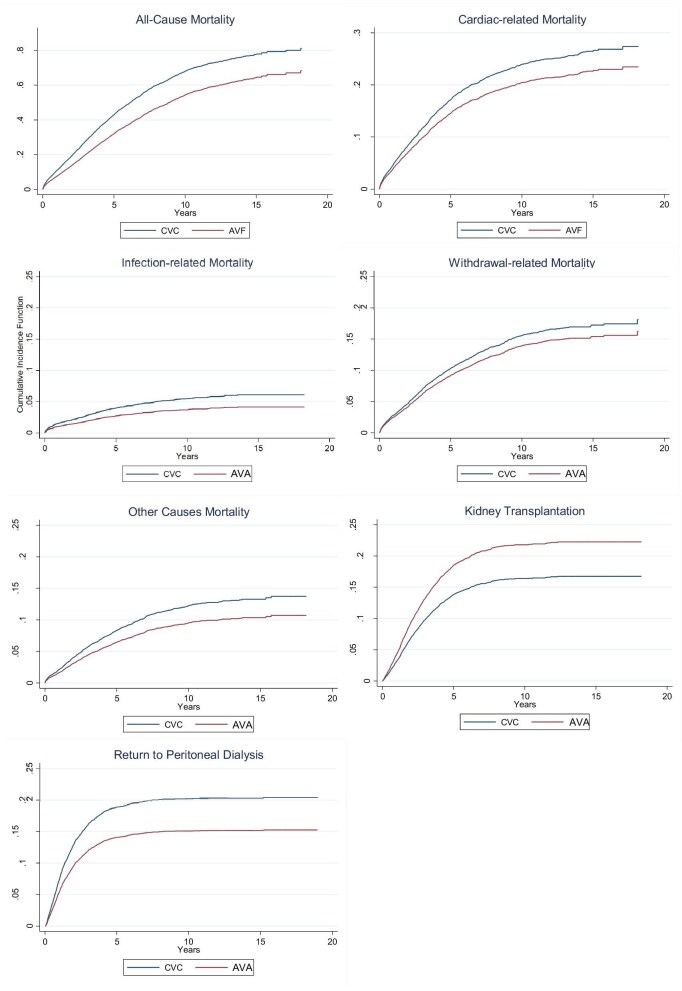
Cumulative incidence function graphs showing time to event by CVC or AVA at HD transfer for (**a**) all-cause mortality, (**b**) cardiac-related mortality, (**c**) infection-related mortality, (**d**) withdrawal-related mortality, (**e**) other causes of mortality, (**f**) kidney transplantation and (**g**) return to PD.

The incidence rates for all-cause and cause-specific mortality are shown in Table 2. All-cause mortality and all the cause-specific mortality rates were significantly lower in the AVA compared with the CVC group. Table 3 shows the risk of all-cause and cause-specific mortality based on the type of vascular access at the time of HD transfer from Cox proportional hazards regressions. The risk of all-cause mortality was lower in patients who transferred to HD with an AVA compared with a CVC (HR 0.71, 95% CI 0.66–0.77), with the risk lowest for infection-related mortality (HR 0.59, 95% CI 45–0.77). Results were consistent in the competing risk analysis (Table 4), with the exception of withdrawal from treatment as a cause of mortality, where no significant effect was seen (SHR 0.89, 95% CI 0.75–1.04). The cumulative incidence curves for all-cause and cause-specific mortality, stratified by access at HD transfer, are shown in Fig. 5.

**Table 2:** Incidence rates of all-cause and cause-specific mortality following HD transfer.

Mortality classification	All study group	AVA	CVC	*P*
All-cause	158 (153–163)	130 (123–139)	175 (168–182)	<.001
Cardiac-related	62 (59–65)	52 (47–57)	68 (64–73)	<.001
Infection-related	16 (14–18)	11 (8–13)	19 (17–21)	<.001
Dialysis withdrawal	49 (46–52)	43 (39–48)	52 (49–56)	.001
Other	32 (29–34)	25 (22–29)	36 (33–39)	<.001

Values are incidence rates (95% CI) per 1000 patient-years, with *P* values for tests of incidence rate difference between AVA and CVC patients.

**Table 3:** Association between access at HD transfer with all-cause and cause-specific mortality, transplantation, and return to PD for Cox proportional hazards regressions (adjusted HR and 95% CI).

	Model 1	Model 2	Model 3	Model 4
Mortality				
All-cause	0.74 (0.68–0.79)	0.73 (0.67–0.79)	0.74 (0.68–0.79)	0.71 (0.66–0.77)
Cardiovascular	0.78 (0.69–0.89)	0.76 (0.68–0.88)	0.78 (0.69–0.89)	0.76 (0.67–0.87)
Infection	0.58 (0.46–0.75)	0.60 (0.47–0.78)	0.61 (0.47–0.79)	0.59 (0.45–0.77)
Withdrawal	0.76 (0.66–0.87)	0.75 (0.65–0.86)	0.75 (0.65–0.87)	0.72 (0.63–0.84)
Other	0.70 (0.59–0.83)	0.68 (0.57–0.81)	0.68 (0.57–0.81)	0.66 (0.56–0.79)
Transplant	1.28 (1.15–1.42)	1.15 (1.02–1.29)	1.14 (1.02–1.28)	1.18 (1.05–1.33)
Return to PD	0.53 (0.47–0.60)	0.55 (0.49–0.63)	0.58 (0.49–0.63)	0.67 (0.59–0.71)

Model 1: unadjusted; Mode 2: Model 1 + age, gender, BMI category, PD vintage, peritonitis, late referral, smoking, and kidney failure cause; Model 3: Model 2 + chronic lung disease, coronary artery disease, peripheral vascular disease, cerebrovascular disease, diabetes, and cancer; Model 4: Model 3 + cause of HD transfer.

**Table 4:** Association between access at HD transfer with all-cause and cause-specific mortality, transplantation, and return to PD for competing risk analysis (adjusted sub-hazard ratio and 95% CI).

	Model 1	Model 2	Model 3	Model 4
Mortality				
All cause	0.69 (0.63–0.74)	0.71 (0.65–0.77)	0.71 (0.66–0.77)	0.69 (0.63–0.75)
Cardiovascular	0.77 (0.69–0.86)	0.83 (0.74–0.93)	0.85 (0.76–0.95)	0.84 (0.75–0.93)
Infection	0.61 (0.49–0.76)	0.68 (0.54–0.85)	0.69 (0.55–0.86)	0.68 (0.54–0.85)
Withdrawal	0.85 (0.72–1.02)	0.90 (0.77–1.07)	0.91 (0.77–1.06)	0.89 (0.75–1.04)
Other	0.74 (0.59–0.91)	0.77 (0.61–0.96)	0.77 (0.61–0.96)	0.77 (0.61–0.97)
Transplant	1.55 (1.34–1.79)	1.36 (1.17–1.57)	1.32 (1.15–1.52)	1.37 (1.19–1.58)
Return to PD	0.56 (0.45–0.69)	0.62 (0.51–0.75)	0.62 (0.51–0.75)	0.72 (0.60–0.87)

Model 1: unadjusted; Mode 2: Model 1 + age, gender, BMI category, PD vintage, peritonitis, late referral, smoking and kidney failure cause; Model 3: Model 2 + chronic lung disease, coronary artery disease, peripheral vascular disease, cerebrovascular disease, diabetes and cancer; Model 4: Model 3 + Cause of HD transfer.

### Association of access at haemodialysis transfer with kidney transplantation and return to peritoneal dialysis

During follow-up, 1481 (22%) patients received a kidney transplant, with a higher proportion among patients starting with an AVA compared with those starting with a CVC (28% versus 18%, *P *< .001). A kidney transplant was more likely to occur in patients with an AVA compared with those with a CVC (HR 1.18, 95% CI 1.05–1.33) (Table 3). Results of the competing risk analysis are shown in Table 4 and Fig. 5.

Following transfer to HD, 1504 (22%) patients returned to PD. A significantly higher proportion of patients with a CVC returned to PD compared with those with an AVA (26% versus 16%, *P *< .001). Patients who transferred to HD with an AVA had a significantly lower likelihood of returning to PD compared with those with a CVC (HR 0.67, 95% CI 0.59–0.71) (Table 3), with similar estimates seen in the competing risk analysis (Table 4, Fig. 5).

### Sensitivity analysis

We conducted two sensitivity analyses for patients who remained on HD after >30 days and >90 days post-HD transfer. For the first sensitivity analysis (including only patients remaining on HD after transfer for >30 days) we included 6546 patients, with the baseline characteristics of the whole group and AVA and CVC groups presented in Supplementary Table S1. For the second sensitivity analysis (including only patients remaining on HD after transfer for >90 days) we included 6222 patients, with the characteristics of the groups presented in Supplementary Table S2.

The risk of all-cause mortality, cause-specific mortality, kidney transplantation, and return to PD between the AVA and CVC groups were comparable to those in the original analysis for both the Cox proportional hazards regressions and the competing risk analysis (Supplementary Tables S3–S6, Supplementary Figs S1 and S2).

### Interaction tests

Interaction tests demonstrated a significant association between cause of kidney failure and cause of HD transfer for all-cause mortality and return to PD (Supplementary Table S7). Subgroup analyses stratified by kidney failure cause and cause of HD transfer are summarized in Supplementary Table S8.

## DISCUSSION

Our longitudinal observation cohort study demonstrated that for PD patients transferring to HD an AVA as the vascular access compared with a CVC was associated with better outcomes for mortality, cause-specific mortality and kidney transplantation. However, returning to PD was more likely in patients transferring to HD with a CVC compared with an AVA. Similar results were found in the sensitivity analysis, where patients who had been on HD for <30 days and <90 days had been excluded.

There is a paucity of studies examining outcomes for PD patients according to vascular access at HD transfer. Only one study conducted more than a decade ago examined this association with mortality and transplantation outcomes [17]. A similar result was observed in that study, with an AVA resulting in a lower risk of mortality; however, only a borderline significance for an increase in kidney transplantation with an AVA was seen. This was likely due to the smaller number of included patients and shorter follow-up. Our analysis also added novel information by examining cause-specific mortality, showing a disproportionate increase in the risk of infection-related mortality for HD transfer with a CVC compared with an AVA. We also showed a significant likelihood of kidney transplantation with HD transfer with an AVA compared with a CVC. No previous studies examined the likelihood of a kidney transplantation according to vascular access in the HD population or in the PD population who transfer to HD, and this association should be examined further.

For patients on HD the optimal vascular access, where possible and feasible, is an AVA. This recommendation is emphasized in numerous guidelines, including Caring for Australasians and New Zealanders with Kidney Impairment (CARI) [18], the Canadian Society of Nephrology (CSN) [19], the United Kingdom Kidney Association [20], and the Kidney Dialysis Outcome Quality Initiative (KDOQI) [10]. Advantages of an AVA over a CVC include a reduction in the risk of infection-related hospital admissions and central vein stenosis [21, 22], and improved blood flow and clearance [22]. A CVC can also be detrimental to vascular health, as evidenced through the observation that AVA use in patients with a previous CVC insertion has poorer outcomes [23]. One Australian study also showed an 80% increased risk of AVAs failing to mature in patients with a previous CVC [24].

These guidelines and studies strongly advocate and support the use of an AVA over a CVC in patients who commence HD. However, the data are primarily based on incident HD patients commencing HD as the first kidney replacement modality. While it is anticipated that the information from HD studies and guidelines, based on incident HD, are generalizable and transferrable for applicability to the PD population, studies with PD patients are still required to confirm this association. Generalizing the information from HD studies to PD patients may result in suboptimal treatment for patients on PD, without first confirming the association. This study shows that the outcomes appear to be consistent for PD patients who transfer to HD with an AVA compared with a CVC, similar to the findings observed in incident HD patients.

We found a large variability in Australia and New Zealand in vascular access used on HD transfer. Some centres transferred only 13% of PD patients to HD with a CVC, compared with others, which could reach a proportion of 98%. Factors that could contribute to this large variability include resource availability, such as a lack of access to vascular surgeons for timely creation of an AVA or the lack of a vascular access nurse for follow-up, and care of AVA health, in addition to other unmeasurable confounders. However, it is also anticipated that the absence of guidelines, clear recommendations, and evidence to support the practice of vascular access for PD patients who transfer to HD likely contributes to the large variability. Our findings can be used to encourage centres to consider strategies to optimize HD transfer with an AVA, if feasible.

The question of optimal vascular access for PD patients in anticipation of HD transfer is complex. While our current study confirms that patients who transfer to HD with an AVA have better outcomes than those who transfer with a CVC, there is a lack of clarity on the timing of AVA creation or the optimal approach. HD transfer will affect almost half of patients on PD in Australia [25], with the high rates of HD transfer compounded by the low rate of return to PD [14]. In addition, most cases of HD transfer are acute, such as due to an infectious cause, which alone accounts for almost half of the HD transfers [25]. The urgency of HD transfer may not allow time to create an AVA, which may explain the high proportion of patients who transfer to HD with a CVC. An alternative option to prevent HD transfer with a CVC would be the approach of creating a backup arterio-venous fistula, a potential strategy that may provide a readily available vascular access for potential acute transfers. The use of a backup arterio-venous fistula (bAVF) fistula has been reported in the literature for more than 40 years [26, 27] and its use is associated with a lower risk of transferring to HD with a CVC [28]. However, to date there are no recommendations or guidelines for its creation or use, nor a consensus approach or framework on how to implement this practice.

We anticipated that an AVA would reduce the risk of mortality and increase the likelihood of kidney transplantation in PD patients who transfer to HD. However, the lower likelihood of returning to PD is striking, even after our sensitivity analysis, which was designed to account for short HD transfers from acute causes with a CVC. We postulate that patients with a functioning AVA may achieve better flows and better clearance and exhibit less symptoms than those who transfer with a CVC. This may encourage them to remain on HD compared with patients who transfer with a CVC, who may seek to return to PD access. In our subgroup analysis, we noted that patients were less likely to return to PD if the cause of their transfer to HD was inadequate PD or patient-related factors. On the other hand, patients were more likely to return to PD if their transfer to HD was due to an abdominal wall defect, abdominal surgery, or catheter problems. This information can be used to guide practice regarding which patient group may benefit more from an AVA creation versus CVC insertion. However, we caution that our results and conclusions should be further explored and clarified in future studies that specifically examine these outcomes as primary endpoints.

In our study we used one of the largest national registries to examine the association between transferring to HD from PD with an AVA compared with a CVC, including 6824 patients across Australia and New Zealand. To our knowledge, ours was the first study examining cause-specific mortality and return to PD as outcomes. We used robust statistical methods, including competing risk analysis, to assess these associations. We also performed a sensitivity analysis to account for possible acute reversible HD transfers, which would be more likely to occur with a CVC. Our results were consistent across all analyses.

These strengths should be balanced against the limitations. Our study was retrospective in design and therefore unable to exclude selection bias with the potential for residual confounding even with adjusted analysis. Other unmeasured confounding factors (such as heart failure status, activities of daily living, sociodemographic information, and laboratory data) were not available to be incorporated in the analysis. Our results should therefore be considered as hypothesis-generating, particularly since we cannot account for reverse causality; as an example, older patients with more comorbidities and poorer health are more likely to die earlier but may also be more likely to be assigned to the CVC group, either due to failure of AVA to mature or to concerns with creating an AVA. We were unable to account for any change in vascular access type after the HD transfer start date, which could also represent a bias, and we were unable to audit the data; we are therefore not able to account for the possibility of coding bias. Importantly, we were unable to account for the disadvantages of an AVA creation, including steal syndrome, heart failure, maturation failure (likely contributing to the higher CVC proportion), number of interventions required to assist maturation, and costs. Important sociodemographic information was not available for all patients (such as the New Zealand cohort), and therefore could not be included in the analysis. Finally, we could not determine the timing of AVA creation before HD transfer, or if they were created as backup AVAs, to provide rationale for or against the policy of creating backup AVA.

This study found that patients with an AVA had better overall survival and kidney transplantation rates compared with those with a CVC. However, patients transferring with an AVA were less likely to return to PD. These findings would support AVA creation for PD patients who may require transfer to HD in the future and opens the question of backup AVA creation as a means to avoid a CVC. Our findings are in keeping with results obtained from the HD population. Future research should focus on identifying strategies for increasing uptake of AVA at HD transfer, the timing of AVA creation, and consideration of bAVA creation in this patient group.

## Supplementary Material

sfaf025_Supplemental_File

## Data Availability

All analysis conducted are presented in the article and supplementary material. Further inquiries can be directed to the corresponding author.
