# Supplementary material for: Impact of type of vascular access on clinical outcomes in peritoneal dialysis patients transitioning to haemodialysis: an ANZDATA study
Source: Clin Kidney J. 2025 Jan 25;18(3):sfaf025. doi: 10.1093/ckj/sfaf025 (PMC11883222; doi:10.1093/ckj/sfaf025)
Supplement: sfaf025_Supplemental_File [file sfaf025_supplemental_file.docx]

| **Table S1**: Baseline characteristics of all study group and by first access at HD transfer in sensitivity analysis (transferred to HD for >30 days). | | | |
| --- | --- | --- | --- |
|  | All study group n= 6,546 | Vascular Access | |
|  |  | AVA n= 2,360 (36%) | CVC n=4,186 (64%) |
| Male | 4,034 (62) | 1,584 (67) | 2,450 (59) |
| Age (years) | 61 (15) | 61 (15) | 61 (15) |
| Cause of kidney failure  Diabetic Kidney Disease   Reno-vascular  Glomerulonephritis  Cystic  Other | 2,552 (39)  891 (14)  1,527 (23)  536 (8)  1,025 (16) | 842 (36)  320 (14)  619 (26)  207 (9)  365 (16) | 1,710 (41)  571 (14)  908 (22)  329 (8)  660 (16) |
| Late referral to Nephrologist | 706 (11) | 213 (9) | 493 (12) |
| Smoking Status   Non-smoker  Ex-smoker  Current smoker | 3,020 (47)  2,635 (41)  842 (13) | 1,096 (47)  942 (40)  298 (13) | 1,924 (46)  1,693 (41)  544 (13) |
| BMI Categories   Underweight  Normal  Overweight  Obese | 135 (2)  1,830 (28)  2,258 (35)  2,272 (35) | 40 (2)  637 (27)  823 (35)  844 (36) | 95 (2)  1,193 (29)  1,435 (35)  1,428 (34) |
| Comorbidities  Chronic Lung Disease  Coronary artery disease  Peripheral Vascular Disease  Cerebrovascular Disease  Diabetes mellitus  Cancer | 1,023 (16)  2,022 (31)  1,520 (23)  931 (14)  2,532 (39)  964 (15) | 339 (14)  736 (31)  464 (20)  300 (13)  856 (36)  405 (17) | 684 (16)  1,286 (31)  1,056 (25)  631 (15)  1,676 (40)  559 (13) |
| PD Vintage (years) PD Vintage  =<6 months  >6- 24 months  >24 months | 1.3 [0.5, 2.5]  1,815 (28)  2,505 (38)  2,226 (34) | 1.6 [0.7, 2.8]  511 (22)  906 (38)  943 (40) | 1.2 [0.4, 2.3]  1,304 (31)  1,599 (38)  1,283 (31) |
| Prior PD peritonitis | 3,776 (58) | 1,224 (52) | 2,552 (61) |
| Cause of HD transfer  Infection related  Inadequate PD  Patient factors  Abdominal wall defects  Abdominal surgery  Cather related problems  Unknown/Other | 2,431 (37)  1,309 (20)  791 (12)  475 (7)  384 (6)  613 (9)  543 (8) | 668 (28)  688 (29)  354 (15)  130 (6)  119 (5)  173 (7)  228 (10) | 1,763 (42)  621 (15)  437 (10)  345 (8)  265 (6)  440 (11)  315 (8) |
| Data expressed as number (percentage), mean (standard deviation) or median [intra-quartile range].  HD: Haemodialysis, BMI: body mass index; CVC: Central venous catheter; PD: Peritoneal dialysis | | | |

| **Table S2**: Baseline characteristics of all study group and by first access at HD transfer in sensitivity analysis (transferred to HD for >90 days). | | | |
| --- | --- | --- | --- |
|  | All study group n= 6,222 | Vascular Access | |
|  |  | AVA n= 2,273 (37%) | CVC n=3,949 (63%) |
| Male | 3,836 (62) | 1,524 (67) | 2,312 (59) |
| Age (years) | 61 (15) | 61 (15) | 61 (15) |
| Cause of kidney failure  Diabetic Kidney Disease  Reno-vascular  Glomerulonephritis  Cystic  Other | 2,424 (39)  840 (14)  1,467 (24)  507 (8)  969 (16) | 812 (36)  308 (14)  600 (27)  198 (9)  348 (15) | 1,612 (41)  532 (14)  867 (22)  309 (8)  621 (16) |
| Late referral to Nephrologist | 679 (11) | 208 (9) | 471 (12) |
| Smoking Status   Non-smoker  Ex-smoker  Current smoker | 2,854 (47)  2,522 (41)  800 (13) | 1,054 (47)  910 (41)  285 (13) | 1,800 (46)  1,612 (41)  515 (13) |
| BMI Categories   Underweight  Normal  Overweight  Obese | 130 (2)  1,743 (28)  2,135 (35)  2,166 (35) | 40 (2)  615 (27)  794 (35)  808 (36) | 90 (2)  1,128 (29)  1,341 (34)  1,358 (35) |
| Comorbidities  Chronic Lung Disease  Coronary artery disease  Peripheral Vascular Disease  Cerebrovascular Disease  Diabetes mellitus  Cancer | 963 (16)  1,914 (31)  1,438 (23)  888 (14)  2,395 (39)  921 (15) | 322 (14)  704 (31)  444 (20)  290 (13)  825 (36)  388 (17) | 641 (16)  1,210 (31)  994 (25)  598 (15)  1,570 (40)  533 (14) |
| PD Vintage (years) PD Vintage  =<6 months  >6- 24 months  >24 months | 1.3 [0.5, 2.5]  1,756 (28)  2,382 (38)  2,084 (34) | 1.6 [0.7, 2.8]  494 (22)  880 (39)  899 (40) | 1.2 [0.4, 2.3]  1,262 (32)  1,502 (38)  1,185 (30) |
| Prior PD peritonitis | 3,583 (58) | 1,175 (52) | 2,408 (61) |
| Cause of HD transfer  Infection related  Inadequate PD  Patient factors  Abdominal wall defects  Abdominal surgery  Cather related problems  Unknown/Other | 2,289 (37)  1,249 (20)  750 (12)  457 (7)  366 (6)  592 (10)  519 (8) | 640 (28)  664 (29)  339 (15)  124 (6)  114 (5)  169 (7)  223 (10) | 1,649 (42)  585 (15)  411 (10)  333 (8)  252 (6)  423 (11)  296 (8) |
| Data expressed as number (percentage), mean (standard deviation) or median [intra-quartile range].  HD: Haemodialysis, BMI: body mass index; CVC: Central venous catheter; PD: Peritoneal dialysis | | | |

| **Table S3**: Association between access at HD transfer with all-cause, cause specific mortality, transplantation and return to PD, for patients remaining on HD >30 days for Cox proportional hazards regressions | | | | |
| --- | --- | --- | --- | --- |
|  | Model 1  HR (95%CI) | Model 2  HR (95%CI) | Model 3  HR (95%CI) | Model 4  HR (95%CI) |
| Mortality  All cause  Cardiac  Infection  Withdrawal  Other | 0.77 (0.71- 0.83)  0.82 (0.72- 0.93)  0.62 (0.48- 0.79)  0.77 (0.67- 0.89)  0.72 (0.61- 0.86) | 0.75 (0.69- 0.82)  0.80 (0.71- 0.91)  0.63 (0.48- 0.81)  0.76 (0.66- 0.88)  0.70 (0.58- 0.84) | 0.76 (0.71- 0.83)  0.89 (0.27- 0.93)  0.63 (0.48- 0.83)  0.77 (0.66- 0.89)  0.69 (0.58- 0.84) | 0.74 (0.68- 0.80)  0.79 (0.69- 0.90)  0.59 (0.45- 0.78)  0.74 (0.64- 0.86)  0.69 (0.57- 0.83) |
| Transplant | 1.29 (1.15- 1.43) | 1.16 (1.03- 1.30) | 1.15 (1.02- 1.29) | 1.19 (1.06- 1.34) |
| Return to PD | 0.53 (0.47- 0.60) | 0.55 (0.48- 0.63) | 0.56 (0.49- 0.63) | 0.67 (0.59- 0.76) |
| Data expressed as adjusted hazard ratio and 95%CI.  Model 1: unadjusted; Mode 2: Model 1 + age, gender, BMI category, PD vintage, peritonitis, late referral, smoking and kidney failure cause; Model 3: Model 2 + chronic lung disease, coronary artery disease, peripheral vascular disease, cerebrovascular disease, diabetes and cancer; Model 4: Model 3 + Cause of HD transfer. | | | | |

| **Table S4**: Association between access at HD transfer with all-cause, cause specific mortality, transplantation and return to PD, for patients remaining on HD >30 days, for competing risk analysis. | | | | |
| --- | --- | --- | --- | --- |
|  | Model 1  SHR (95%CI) | Model 2  SHR (95%CI) | Model 3  SHR (95%CI) | Model 4  SHR (95%CI) |
| Mortality  All cause  Cardiac  Infection  Withdrawal  Other | 0.70 (0.65- 0.77)  0.79 (0.70- 0.88)  0.63 (0.49- 0.79)  0.85 (0.71- 1.02)  0.75 (0.59- 0.94) | 0.72 (0.66- 0.79)  0.85 (0.75- 0.96)  0.68 (0.54- 0.86)  0.89 (0.75- 1.05)  0.78 (0.61- 0.99) | 0.73 (0.66- 0.79)  0.86 (0.77- 0.97)  0.69 (0.55- 0.87)  0.89 (0.76- 1.04)  0.78 (0.61- 0.99) | 0.70 (0.64- 0.77)  0.85 (0.76- 0.96)  0.67 (0.53- 0.84)  0.87 (0.74- 1.02)  0.78 (0.60- 1.00) |
| Transplant | 1.53 (1.31- 1.78) | 1.36 (1.17- 1.58) | 1.33 (1.15- 1.54) | 1.38 (1.19- 1.59) |
| Return to PD | 0.54 (0.44- 0.66) | 0.60 (0.49- 0.72) | 0.60 (0.50- 0.73) | 0.71 (0.59- 0.84) |
| Data expressed as adjusted sub-hazard ratio and 95%CI.  Model 1: unadjusted; Mode 2: Model 1 + age, gender, BMI category, PD vintage, peritonitis, late referral, smoking and kidney failure cause; Model 3: Model 2 + chronic lung disease, coronary artery disease, peripheral vascular disease, cerebrovascular disease, diabetes and cancer; Model 4: Model 3 + Cause of HD transfer. | | | | |

| **Table S5**: Association between access at HD transfer with all-cause, cause specific mortality, transplantation and return to PD, for patients remaining on HD >90 days, for Cox proportional hazards regressions. | | | | |
| --- | --- | --- | --- | --- |
|  | Model 1  HR (95%CI) | Model 2  HR (95%CI) | Model 3  HR (95%CI) | Model 4  HR (95%CI) |
| Mortality  All cause  Cardiac  Infection  Withdrawal  Other | 0.78 (0.72- 0.84)  0.82 (0.72- 0.93)  0.63 (0.48- 0.83)  0.80 (0.69- 0.92)  0.72 (0.61- 0.86) | 0.77 (0.71- 0.84)  0.81 (0.72- 0.93)  0.65 (0.49- 0.85)  0.79 (0.68- 0.91)  0.70 (0.58- 0.84) | 0.78 (0.71- 0.84)  0.83 (0.72- 0.94)  0.65 (0.49- 0.86)  0.79 (0.69- 0.92)  0.70 (0.58- 0.84) | 0.75 (0.69- 0.82)  0.80 (0.70- 0.92)  0.61 (0.47- 0.81)  0.77 (0.66- 0.89)  0.69 (0.57- 0.83) |
| Transplant | 1.31 (1.17- 1.46) | 1.19 (1.06- 1.34) | 1.18 (1.05- 1.33) | 1.21 (1.08- 1.37) |
| Return to PD | 0.53 (0.47- 0.59) | 0.55 (0.49- 0.63) | 0.56 (0.49- 0.63) | 0.67 (0.59- 0.76) |
| Data expressed as adjusted hazard ratio and 95%CI.  Model 1: unadjusted; Mode 2: Model 1 + age, gender, BMI category, PD vintage, peritonitis, late referral, smoking and kidney failure cause; Model 3: Model 2 + chronic lung disease, coronary artery disease, peripheral vascular disease, cerebrovascular disease, diabetes and cancer; Model 4: Model 3 + Cause of HD transfer. | | | | |

| **Table S6**: Association between access at HD transfer with all-cause, cause specific mortality, transplantation and return to PD, for patients remaining on HD >90 days, for competing risk analysis. | | | | |
| --- | --- | --- | --- | --- |
|  | Model 1  SHR (95%CI) | Model 2  SHR (95%CI) | Model 3  SHR (95%CI) | Model 4  SHR (95%CI) |
| Mortality  All cause  Cardiac  Infection  Withdrawal  Other | 0.71 (0.65- 0.78)  0.79 (0.69- 0.87)  0.63 (0.49- 0.81)  0.86 (0.72- 1.02)  0.74 (0.59- 0.93) | 0.73 (0.68- 0.80)  0.85 (0.75- 0.96)  0.69 (0.54- 0.89)  0.89 (0.76- 1.06)  0.76 (0.59- 0.98) | 0.73 (0.69- 0.81)  0.86 (0.76- 0.98)  0.70 (0.55- 0.90)  0.89 (0.76- 1.05)  0.76 (0.59- 0.98) | 0.71 (0.65- 0.78)  0.85 (0.75- 0.97)  0.68 (0.53- 0.86)  0.88 (0.75- 1.03)  0.76 (0.59- 0.99) |
| Transplant | 1.54 (1.31- 1.79) | 1.37 (1.18- 1.60) | 1.35 (1.16- 1.57) | 1.39 (1.20- 1.61) |
| Return to PD | 0.52 (0.42- 0.65) | 0.59 (0.48- 0.71) | 0.59 (0.48- 0.71) | 0.69 (0.58- 0.83) |
| Data expressed as adjusted sub-hazard ratio and 95%CI.  Model 1: unadjusted; Mode 2: Model 1 + age, gender, BMI category, PD vintage, peritonitis, late referral, smoking and kidney failure cause; Model 3: Model 2 + chronic lung disease, coronary artery disease, peripheral vascular disease, cerebrovascular disease, diabetes and cancer; Model 4: Model 3 + Cause of HD transfer. | | | | |

| **Table S7**: *P*-value for Interaction tests between vascular access at HD transfer and gender, PD vintage, peritonitis, cause of kidney failure and cause of HD transfer on outcomes. | | | |
| --- | --- | --- | --- |
|  | All- Cause Mortality | Kidney Transplant | Return to PD |
| Gender  PD vintage  Peritonitis  Cause KF  Cause HD transfer | 0.7  0.5  0.6  0.02  0.02 | 0.9  0.1  0.6  0.9  0.7 | 0.2  0.1  0.7  0.01  <0.001 |
|  | | | |

| **Table S8**: Relationship between vascular access on HD transfer and outcomes stratified by cause of kidney failure and cause of HD transfer. | | | |
| --- | --- | --- | --- |
|  | All- Cause Mortality | Kidney Transplant | Return to PD |
|  | HR (95%CI) | HR (95%CI) | HR (95%CI) |
| KF Cause  CVC  Diabetes  Hypertension  Glomerulonephritis  Cystic  Other  AVA  Diabetes  Hypertension  Glomerulonephritis  Cystic  Other | Ref  0.94 (0.82- 1.08)  0.80 (0.69- 0.92)  0.47 (0.37- 0.60)  1.05 (0.92- 1.21)  0.75 (0.67- 0.85)  0.63 (0.52- 0.75)  0.55 (0.47- 0.66)  0.52 (0.39- 0.69)  0.66 (0.55- 0.79) | Ref  0.80 (0.59- 1.09)  1.25 (1.01- 1.53)  1.82 (1.43- 2.32)  0.92 (0.72- 1.17)  1.36 (1.09- 1.70)  1.00 (0.72- 1.39)  1.55 (1.26- 1.92)  2.39 (1.83- 3.10)  1.21 (0.94- 1.59) | Ref  0.92 (0.74- 1.13)  0.84 (0.69- 1.02)  0.96 (0.76- 1.21)  0.99 (0.81- 1.23)  0.63 (0.51- 0.78)  0.64 (0.47- 0.86)  0.70 (0.55- 0.89)  0.93 (0.67- 1.30)  0.53 (0.39- 0.72) |
| Cause of HD transfer  CVC  Infection related  Inadequate PD  Patient factors  Abdominal wall defect  Abdominal surgery  Catheter problems  Unknown/other  AVA  Infection related  Inadequate PD  Patient factors  Abdominal wall defect  Abdominal surgery  Catheter problems  Unknown/other | Ref  1.08 (0.94- 1.24)  1.38 (1.20- 1.59)  0.76 (0.64- 0.92)  0.77 (0.64- 0.93)  0.97 (0.84- 1.13)  1.24 (1.04- 1.47)  0.75 (0.66- 0.85)  0.71 (0.62- 0.82)  0.89 (0.76- 1.05)  0.59 (0.44- 0.79)  0.97 (0.73- 1.29)  0.64 (0.49- 0.82)  0.83 (0.67- 1.04) | Ref  1.01 (0.82- 1.25)  0.47 (0.34- 0.66)  1.07 (0.84- 1.37)  1.15 (0.88- 1.51)  0.89 (0.69- 1.14)  0.95 (0.71- 1.25)  1.37 (1.13- 1.66)  1.16 (0.96- 1.41)  0.75 (0.57- 0.99)  1.36 (0.99- 1.88)  1.30 (0.90- 1.88)  1.19 (0.88- 1.63)  1.33 (1.01- 1.76) | Ref  0.43 (0.32- 0.59)  0.40 (0.28- 0.57)  2.31 (1.93- 2.76)  1.89 (1.55- 2.31)  1.87 (1.56- 2.25)  1.24 (0.95- 1.61)  0.58 (0.47- 0.72)  0.24 (0.17- 0.34)  0.41 (0.29- 0.59)  1.74 (1.31- 2.31)  2.87 (2.21- 3.74)  1.11 (0.82- 1.50)  0.43 (0.27- 0.69) |
|  | | | |
